# Defining neuronal responses to the neurotropic parasite *Toxoplasma gondii*

**DOI:** 10.1128/msphere.00216-25

**Published:** 2025-05-30

**Authors:** Hannah J. Johnson, Joshua A. Kochanowsky, Sambamurthy Chandrasekaran, Christopher A. Hunter, Daniel P. Beiting, Anita A. Koshy

**Affiliations:** 1 Neuroscience Graduate Interdisciplinary Program, University of Arizona 8041, Tucson, Arizona, USA; 2 BIO5 Institute, University of Arizona 124486, Tucson, Arizona, USA; 3 Department of Immunobiology, University of Arizona 242724, Tucson, Arizona, USA; 4 Department of Pathobiology, School of Veterinary Medicine, University of Pennsylvania 634332, Philadelphia, Pennsylvania, USA; 5 Department of Neurology, University of Arizona 218526, Tucson, Arizona, USA; Virginia-Maryland College of Veterinary Medicine, Blacksburg, Virginia, USA

**Keywords:** *Toxoplasma gondii*, *T. gondii*, neurons, RNA-seq, transcriptomics, host response, central nervous system infections

## Abstract

A select group of pathogens infects neurons in the brain. Prior dogma held that neurons were “defenseless” against infecting microbes, but many studies suggest that neurons can mount anti-microbial defenses. However, a knowledge gap in understanding how neurons respond *in vitro* and *in vivo* to different classes of microorganisms remains. To address this gap, we compared a transcriptional data set derived from primary neuron cultures (PNCs) infected with the neurotropic intracellular parasite *Toxoplasma gondii* with a data set derived from neurons injected with *T. gondii* protein *in vivo*. These curated responses were then compared to the transcriptional responses of PNCs infected with the single-stranded RNA viruses, West Nile virus or Zika virus. These analyses highlighted a conserved response to infection associated with chemokines (*Cxcl10, Ccl2*) and cytokines (interferon signaling). However, *T. gondii* had diminished IFN-α signaling *in vitro* compared to the viral data sets and was uniquely associated with a decrease in neuron-specific genes (*Snap25*, *Slc17a7*, *Prkcg*). These data underscore that neurons participate in infection-induced neuroinflammation and illustrate that neurons possess both pathogen-specific and pathogen-conserved responses.

**IMPORTANCE:**

Though neurons are commonly the target of pathogens that infect the central nervous system (CNS), few data sets assess the neuronal response to infection. This paucity of data is likely because neurons are perceived to have diminished immune capabilities. However, to understand the role of neurons in neuroinflammation and their immune capabilities, their responses must be investigated. Here, we analyzed publicly accessible, neuron-specific data sets to compare neuron responses to a eukaryotic pathogen vs two Orthoflaviviruses. A better understanding of neuron responses to different infections will allow us to develop methods for inhibiting pathways that lead to neuron dysfunction, enhancing those that limit pathogen survival, and mitigating infection-induced damage to the CNS.

## INTRODUCTION

A select number of microbes (e.g., measles virus and *Toxoplasma gondii*) infect the central nervous system (CNS). For many of these infections, neurons are the CNS cell that is primarily infected (1–3). Until relatively recently, dogma suggested this neuronal predominance arose from neurons lacking cell-intrinsic immune responses. Over several decades, work focusing on viral-neuron interactions established that neurons have cell-intrinsic responses, though these responses can differ from other cell types and even between neuron subtypes (4–7). These studies raise the question of whether the cellular immunity of neurons varies by context and/or pathogen. The eukaryotic intracellular parasite *Toxoplasma gondii* is a non-viral microbe with a tropism for neurons (8) and a broad natural host range, including rodents and humans (9). During infection, parasites invade the CNS where they can infect multiple cell types, but in neurons, a portion of parasites switch to a slow-growing stage that forms tissue cysts (1, 10, 11). These tissue cysts cause a persistent, asymptomatic infection, potentially for the lifetime of the host (10–12). Recent studies suggest that the immune system can recognize infected neurons, contributing to local control of *T. gondii in vivo* (13–16). *In vitro* studies have shown that neurons can be activated by IFN-γ to limit parasite growth (17). Like most intracellular microbes, all of which depend upon the host cell for survival, *T. gondii* highly manipulates its host cell through the secretion of effector proteins. Most of the studies that define how these effector proteins manipulate cells were done *in vitro* in fibroblasts and immune cells such as macrophages (18, 19). While such studies have revealed fundamental aspects of *T. gondii*-host cell biology, they will have missed neuron-specific effects or effects only triggered during *in vivo* infection. The importance of understanding these nuances is highlighted by studies showing that outcomes of *T. gondii*-host cell interactions can vary by *T. gondii* strain and host cell (20–23).

We previously tried to address this gap by using laser capture microdissection (LCM) in combination with our *T. gondii*-Cre system (24). In this system, we use parasites that express a *T. gondii*::Cre recombinase fusion protein (ROP::Cre) to infect Cre reporter mice that express a green fluorescent protein (GFP) only after Cre-mediated recombination. Because the ROP::Cre protein is introduced into the host cell concomitantly with other early effectors (ROPs) and before full invasion, neurons injected with the ROPs will express GFP even if they cleared the parasite or were never invaded (i.e., aborted invasion) (25–27). We then used LCM and RNA-seq to isolate, pool, and transcriptionally profile the somas of *T. gondii*-injected (GFP^+^) neurons (TINs). Though a small area centered on TINs’ somas was captured, these transcriptional data still contained immune cell transcripts (24), making it difficult to distinguish which differentially expressed genes (DEGs) or pathways were derived from neurons, immune cells, or both.

In this study, we sought to identify neuron-specific responses to *T. gondii* by comparing RNA-seq data sets from our *in vivo* data with a newly generated *in vitro* data set from *T. gondii*-infected primary neuron cultures (PNCs). This analysis revealed a set of conserved pathways driven by chemokines, such as *Ccl2* and *Cxcl10*. The comparison to previously published transcriptomes of West Nile virus (WNV)-infected and Zika virus (ZKV)-infected PNCs (7) revealed pathways that were conserved between these data sets and others that were pathogen dependent. For example, *T. gondii* data sets revealed a decrease in neuron-specific genes (e.g., *Snap25*, *Slc17a7*, and *Prkcg*) that were unchanged in virally infected neurons. Conversely, the type I IFN (IFN-α) response pathway was upregulated by WNV and ZKV and, to some extent, by *T. gondii in vivo* but not by *T. gondii in vitro*. In summary, the ability to compare the *in vitro* and *in vivo* response of neurons to infection highlights that neurons have intrinsic, microbe-specific responses that are modulated *in vivo*.

## RESULTS

### Conserved neuron response genes and pathways in *T. gondii* infection models

**Fig 1:**
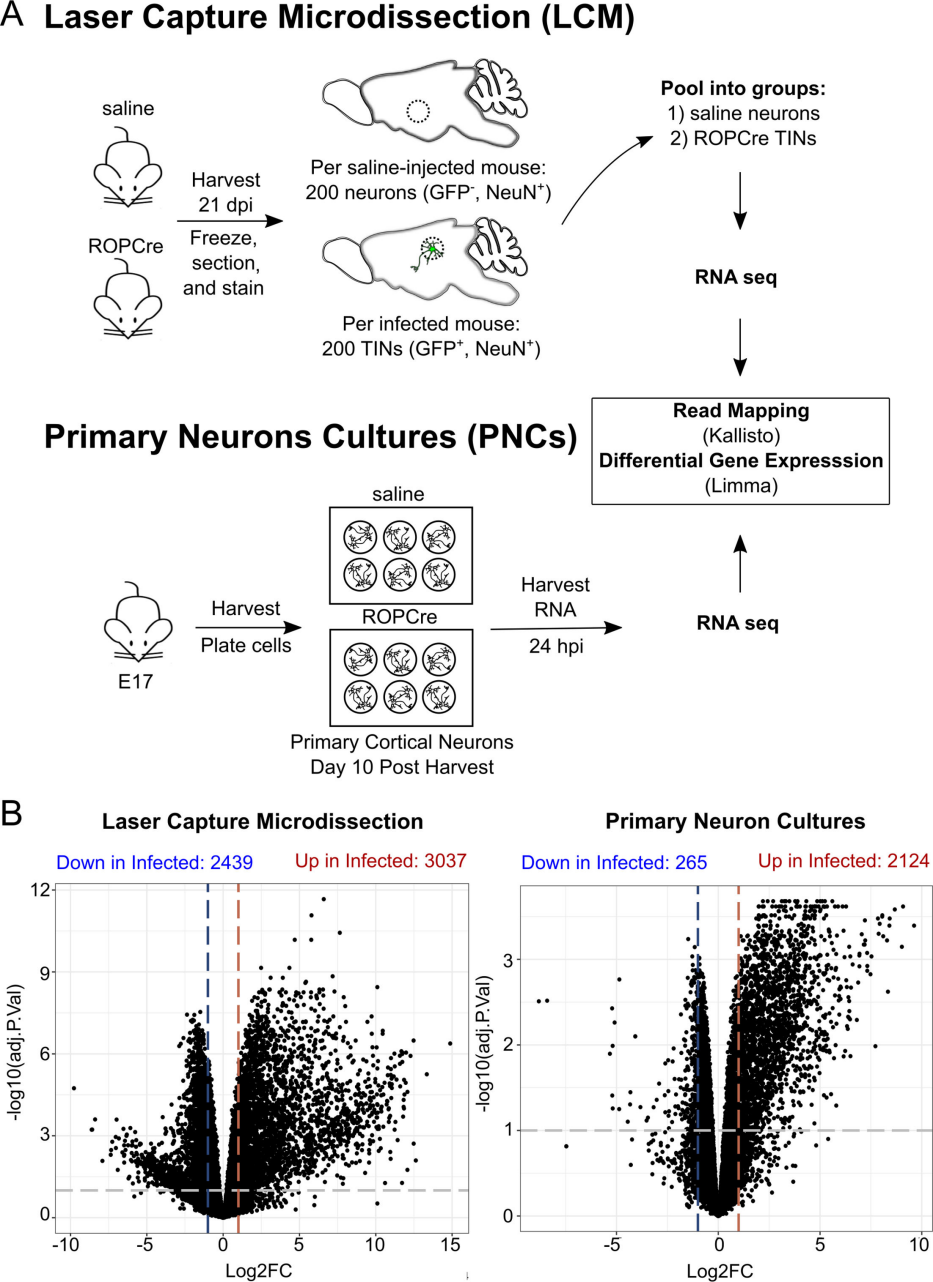
*T. gondii*-infected neurons from *in vitro* (PNC) and *in vivo* (LCM) systems were captured and analyzed for differentially expressed genes. (**A**) Experimental schematic of neurons captured by laser capture microdissection and infected primary murine neuronal cultures. (**B**) Volcano plots of differentially expressed genes in both data sets. Horizontal bars indicate adjusted *P* values ≤0.1, and vertical bars indicate log_2_ fold change ≥1 for up- and downregulated genes.

Transcriptomic data from neurons in two *T. gondii* infection conditions—neurons in tissue sections isolated with laser capture microdissection (24) and cortical primary neuron cultures (Fig. 1)—were compared to find common neuron response pathways. Briefly, in a previous report, we combined transgenic parasites that secrete Cre recombinase with a mouse strain that expresses a Cre-sensitive GFP reporter, which allowed us to isolate *T. gondii*-injected neurons (GFP^+^NeuN^+^) by LCM (27). The RNA from these neurons was isolated, sequenced, and compared with neurons from uninfected mice (Fig. 1A).

In a separate study, PNCs infected with *T. gondii* for 24 hours were used to assess how infection altered the neuronal transcriptome. Reads were filtered, normalized, and represented as counts per million, shown in Fig. S1A through C. As expected, principal component analysis of the LCM and PNC data sets revealed marked differences between infected and uninfected controls (Fig. S1D and E), with over 2,100 differentially expressed genes compared to uninfected controls (false discovery rate [FDR] ≤ 0.1, log_2_ fold change [FC] ≥ 1) (Fig. 1B; Tables S1 and S2).

**Fig 2:**
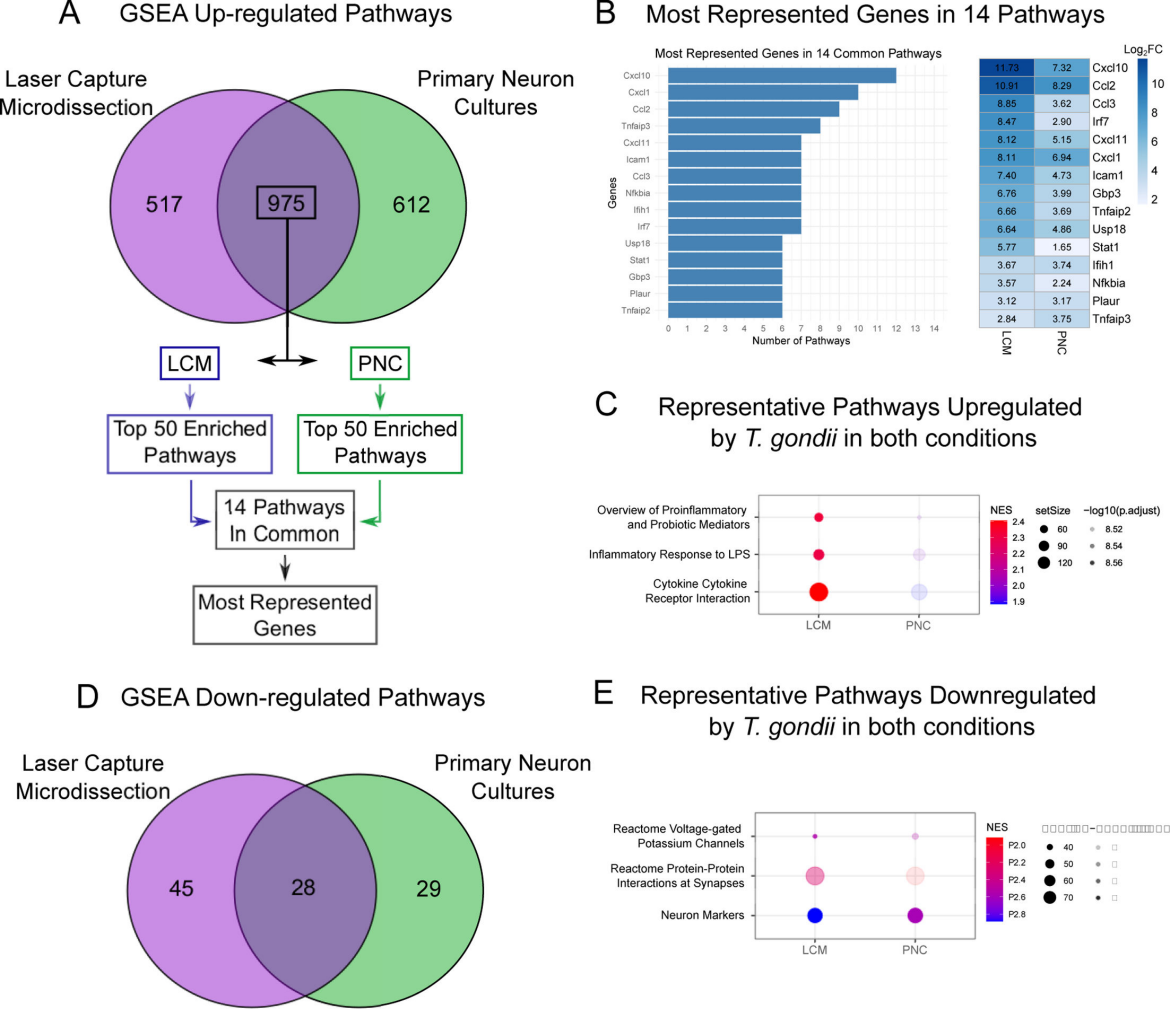
Pathway analysis reveals the neuronal response to *T. gondii* infection involves an increase in proinflammatory cytokines and a decrease in neuron function. (**A**) The top 14 enriched pathways between LCM data set and PNCs were selected out of 975 upregulated pathways. (**B**) Quantification of the most represented genes in the 14 most enriched pathways with a heatmap of their log_2_FC. (**C**) Representative signature pathways between PNCs and LCM with normalized enrichment scores (NES). (**D**) Venn diagram of 28 downregulated pathways in LCM and PNC data sets. (**E**) Enrichment scores of downregulated neuron pathways in *T. gondii* data sets. GSEA, gene set enrichment analysis.

As noted above, the transcriptomes from TINs contained transcripts classically associated with immune cells (24). Such transcripts were not observed in PNCs (Fig. S2), except for *Cd80* and *Cd44,* which are receptors that are expressed during neuron development (28, 29). Thus, the pathways identified in the PNC data that are also identified in the LCM data likely represent neuron-specific responses to *T. gondii*. Functional enrichment analysis of both sample types revealed 975 upregulated pathways shared between infected PNCs and neurons captured *in vivo* (Fig. 2A). A comparison of the top 50 enriched pathways of each condition (LCM or PNCs) identified 14 pathways in common (Table S3). These pathways were associated with responses to different microbial stimuli (e.g., LPS, COVID, and respiratory syncytial virus) and cytokine signaling. By analyzing the individual genes associated with these pathways, we identified a small group of genes that were consistently enriched in these 14 pathways. These genes included CXC motif chemokine ligand 10 (*Cxcl10*, 12 of the 14 pathways), chemokine (C-X-C motif) ligand 1 (*Cxcl1*, 10 of the 14), and chemokine (C-C motif) ligand 2 (*Ccl2*, 9 of the 14) (Fig. 2B). These pathways were functionally similar in that they primarily centered around chemokine/cytokine signaling and proinflammatory responses (Fig. 2C). While both PNCs and the LCM data showed an enrichment of these pathways, the LCM data set showed a higher number of genes involved (set size) and increased log_2_FC of the DEGs (Fig. 2C). Only *Ifih1*, *Plaur*, and *Tnfaip3* were highly represented genes that were equivalent or higher in PNCs vs LCM neurons.

A comparison of the downregulated pathways between LCM and PNCs found 28 pathways in common (Fig. 2D). Many of these pathways were neuron-specific, including neuronal markers, protein-protein interactions at synapses (e.g., SNARE proteins), long-term potentiation, activation of NMDA receptors, and GABA synthesis and receptor signaling. We had previously noticed fewer neuron markers in our LCM data set but could not determine if this decrease was due to an increase in contaminating immune cells comprising a higher proportion of our transcripts or a true decrease in neuronal transcription. However, in the PNCs—which lack immune cells—we still saw a decrease in these neuron-specific pathways and related genes (Table S4). In addition to synaptic and neuron marker pathways, multiple voltage-gated potassium channels were downregulated (Fig. 2E). Using the genes from the GO pathway GOMF_POTASSIUM_ION_LEAK_CHANNEL_ACTIVITY, we found that many were downregulated in both of our data sets (Fig. S3) except for Kcnk5/TASK-2, which was upregulated in both paradigms. In summary, the ability to compare the *in vivo* and *in vitro* data sets appears to be a feasible way to identify neuron-specific responses from complex *in vivo* transcriptional studies and suggests that *T. gondii* may directly modulate neuronal function.

### Comparison of neuronal responses to *T. gondii* or viral infection

**Fig 3:**
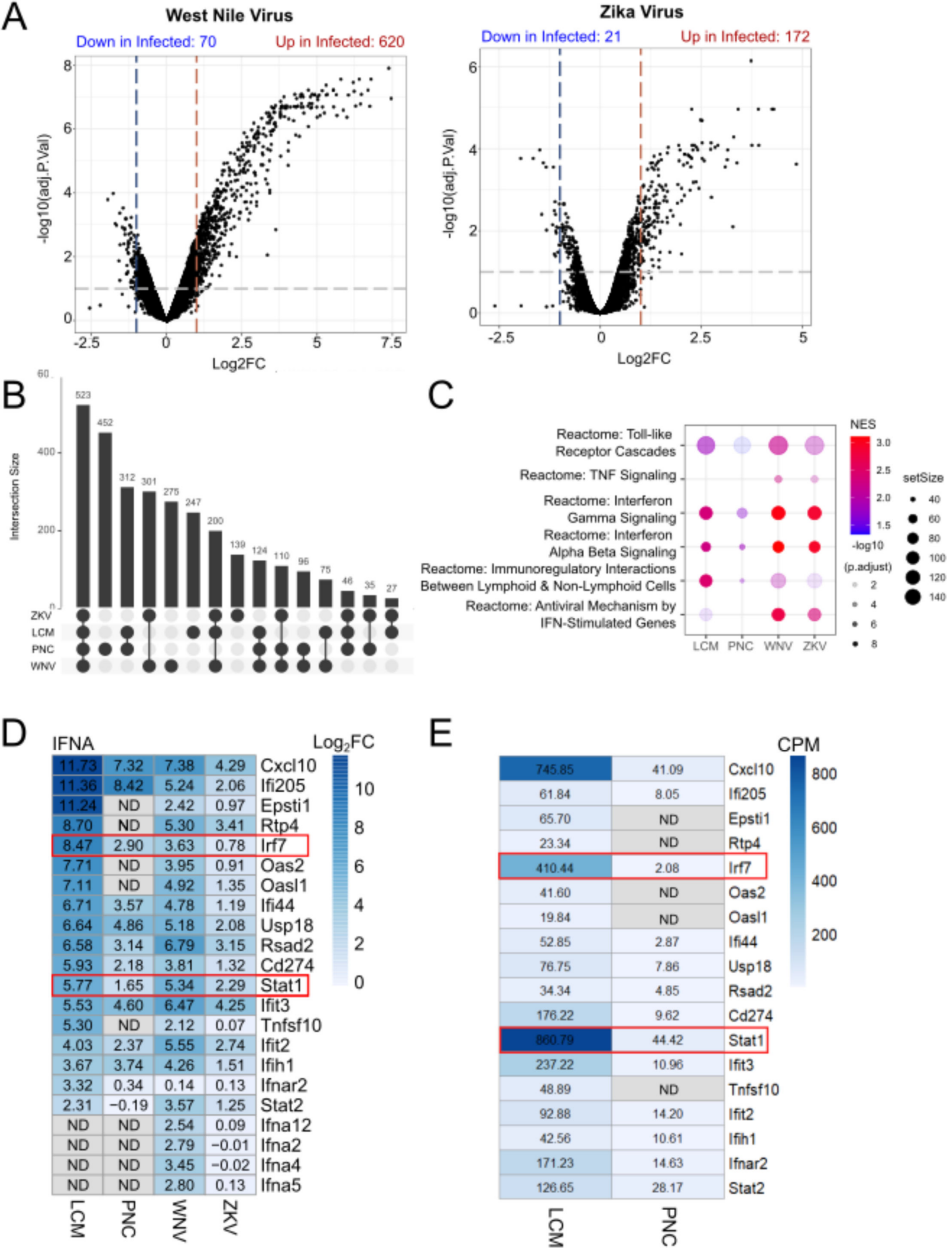
WNV- and ZKV-infected PNCs and the LCM data set show the upregulation of IFN*-α* response genes, unlike *T. gondii*-infected PNCs. (**A**) Volcano plots of WNV and ZKV-infected PNCs. Horizontal bars indicate adjusted *P* values ≤ 0.1, and vertical bars indicate log2 fold change ≥ 1 for up- and downregulated genes. (**B**) Upset plot of upregulated gene set enrichment analysis pathways. (**C**) Relative expression of inflammatory pathways across data sets. (**D**) IFN-α response genes expressed in LCM, *T. gondii*, WNV, and ZKV infected in log_2_FC. (**E**) Count per million of IFN-α response genes in LCM and *T. gondii* PNC data sets with raw values shown. ND, not detected. ND genes in both LCM and PNCs in panel **D** are not included in panel **E**.

To determine if these neuron responses were specific to *T. gondii* or occurred with other relevant infections, we wanted to compare the LCM and PNC data sets with transcriptional studies from neurons infected with non-parasitic microbes. A search of the Gene Expression Omnibus (GEO) repository, the NIH’s publicly funded genomics data repository, identified several transcriptional studies on infected wild-type murine neurons (7, 30–32). From these studies, we analyzed four data sets: Zika virus- and West Nile virus-infected PNCs profiled by microarray (7) and two single-cell RNA-seq studies of cortical neurons infected with a circuit tracing, attenuated rabies virus (31, 32). The latter two data sets (31, 32) had very few genes that met our criteria for DEGs (FDR ≤ 0.1, log_2_FC ≥ 1) and were excluded from further analysis. However, the data sets for ZKV and WNV (7) contained 193 and 690 DEGs, respectively (Fig. 3A), making them amenable for comparison with the *T. gondii* data sets.

Between these data sets, there were 532 pathways in common (Fig. 3B). We further narrowed our focus to pathways relating to IFN-γ, IFN-α/β, and TNF signaling to see if there were differences in these responses between infections (Fig. 3C). We found that all data sets had an enrichment for the IFN-γ signaling pathway and innate immunity pathways (represented by Toll-like receptor cascades) (Fig. 3C), but only the viral data sets showed enrichment for TNF signaling. As expected, the viral data sets showed an enrichment in anti-viral, type I IFN pathways, specifically in IFN-stimulated genes. Consistent with prior work (33), *T. gondii*-infected PNCs showed very little type I IFN response, while the LCM data set showed some enrichment in the “Antiviral Mechanism by IFN-Related Antiviral Mechanisms” pathway (Fig. 3C; Fig. S4). To understand these differences, we compared the individual genes involved in type I IFN pathways and found that several key IFN-α genes were differentially expressed (Fig. 3D and E). *T. gondii* PNCs showed upregulation in *Stat1* and *Irf7* but not in many downstream genes. These downstream genes fell into two clusters, with the first cluster (e.g., *Oas2*, *Oas1l*, and *Epsti1*) showing no baseline expression or upregulation and the second cluster (e.g., *Ifnar2* and *Stat2*) showing low baseline expression and no upregulation (Fig. 3E). WNV, ZKV, and, to a lesser extent, *in vivo* infection with *T. gondii* showed the upregulation of many of the genes in this pathway, though differences could be seen even between these three experimental conditions (e.g., *Ifna2, 4, 5, 12*) (Fig. 3D). Collectively, these data suggest that *in vitro*, WNV and ZKV trigger neuron IFN-α responses, but *T. gondii* does not, while *T. gondii* triggers a broader immune response *in vivo*.

## DISCUSSION

Here, we sought to define how neurons respond to *T. gondii* and determine how this response compares to infection with other neurotropic microbes. To accomplish this goal, we compared four transcriptional data sets: *T. gondii*-injected neurons captured by LCM from infected murine brain tissue and primary murine cortical neuron cultures infected with *T. gondii*, WNV, or ZKV. These comparisons revealed that cortical neurons have conserved responses to these infections but also show key differences that distinguish responses to a virus vs a eukaryotic parasite.

**Fig 4:**
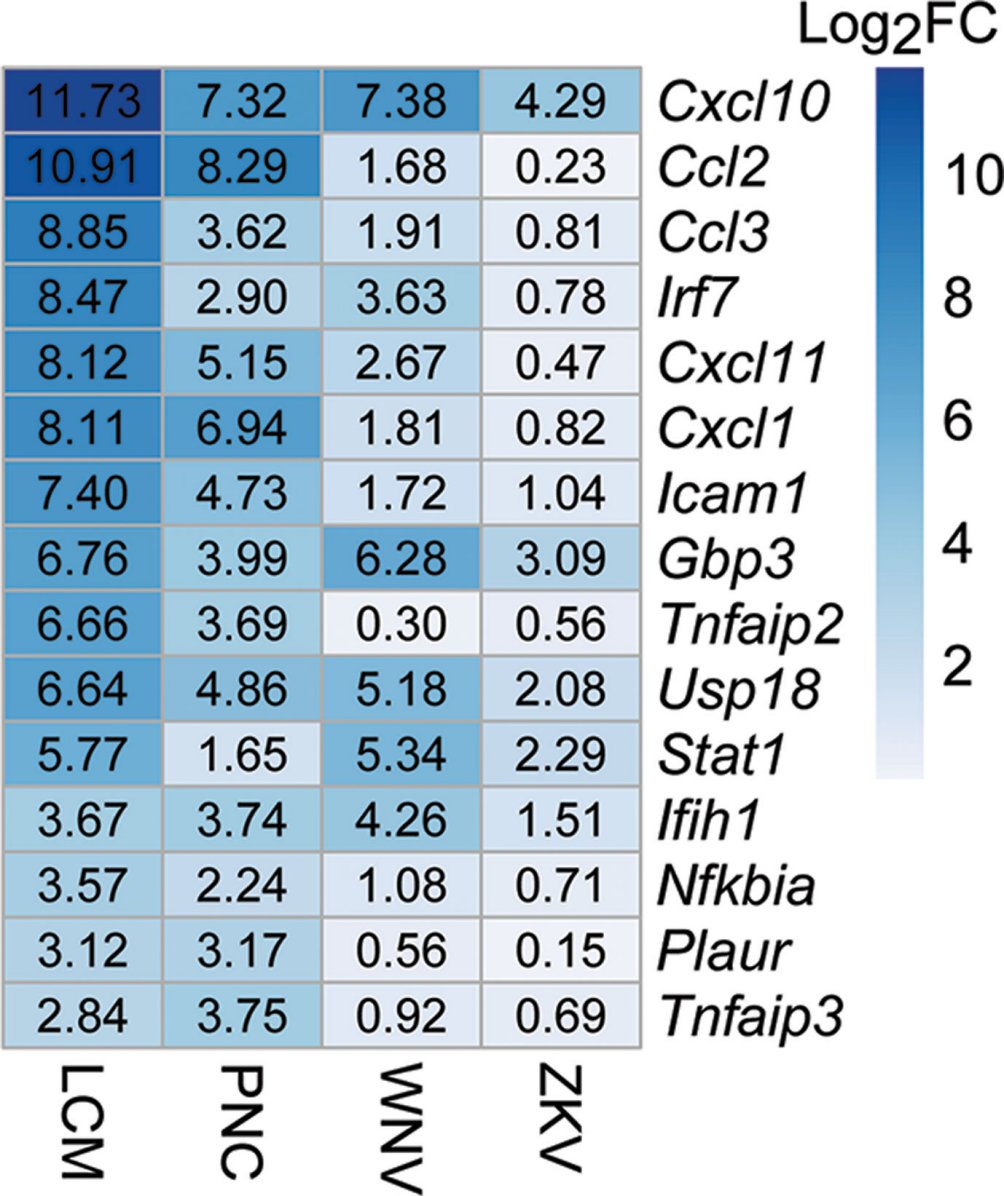
*Cxcl10* is upregulated across acute and subacute data sets. Other conserved genes include *Irf7*, *Icam1*, *Gbp3*, *Usp18*, *Stat1*, and *Ifih1.*

All four data sets had a pronounced increase in inflammatory pathways, including in type I and type II interferon signaling (Fig. 3C). Of the many cytokines/chemokines upregulated in these data sets, *Cxcl10* is highly represented in the pathways upregulated in the LCM and *T. gondii*-infected PNC data sets and is also upregulated in WNV- and ZKV-infected PNCs (Fig. 4). These data suggest that *Cxcl10* upregulation is a conserved feature of the neuron response to these infections. As *Cxcl10* is a chemokine that attracts innate and adaptive immune cells, its conserved upregulation is consistent with the need to attract immune cells to infected neurons, whether the infecting microbe is viral or parasitic (e.g., effector T cells for *T. gondii* [34]). Validating the role of neuronal *Cxcl10* and other key genes/pathways in the outcomes of CNS infection will be the focus of future work.

The differences between the data sets are also revealing. Only the *T. gondii* infection data sets showed a consistent downregulation of neuronal function pathways (markers, long term potentiation [LTP], synapse function, and potassium channels). The downregulation in potassium channels was of particular interest to us because it could explain our recent finding that TINs have a depolarized resting membrane potential when compared to non-injected neighboring neurons or neurons in uninfected mice (35). Neuronal dysfunction associated with *T. gondii* infection has been identified previously (36–41), but the experimental setups made it a challenge to distinguish the direct effect of *T. gondii* on neurons vs effects from infiltrating immune cells or microglia and astrocytes. The findings presented here suggest that *T. gondii* can directly induce neuronal dysfunction.

**Fig 5:**
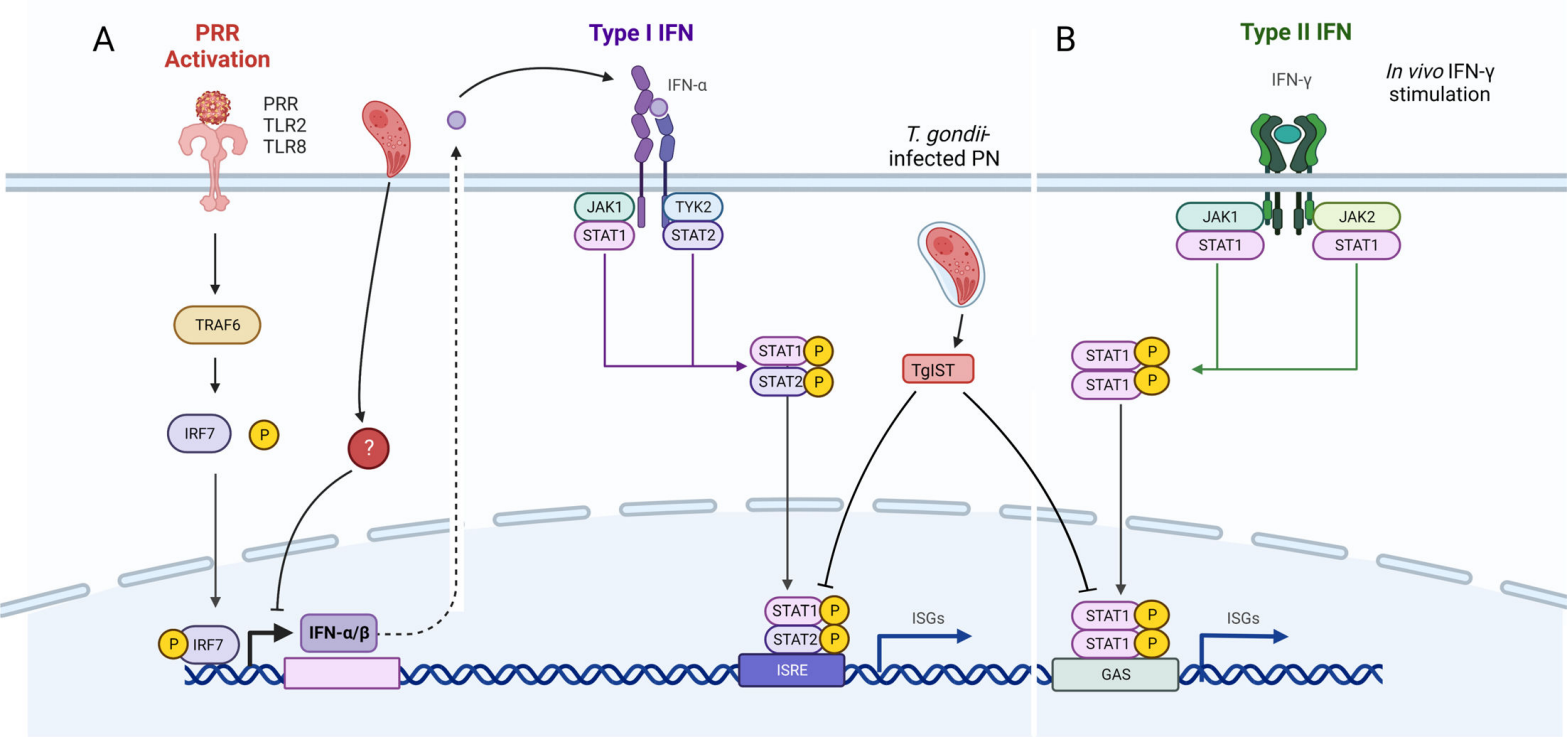
Despite *Irf7* upregulation in all data sets, *T. gondii-*infected neurons fail to upregulate and secrete IFN-α. (**A**) WNV and *T. gondii* activate PRR receptors leading to an intracellular cascade that results in IFN-α production through IRF7 phosphorylation. IFN-α binds IFNAR and upregulates *Stat1* and other subsequent IFN-α response genes in WNV-infected neurons. *T. gondii* inhibits *Stat1* in this pathway *in vitro* with the *T. gondii* effector protein TgIST. The lack of *Ifn-α* upregulation in both *T. gondii* data sets indicates that the parasite may inhibit the action of phosphorylated IRF7 through an unknown mechanism, either before full invasion or after. (**B**) *In vivo, Stat1* may be upregulated through alternative stimulation pathways, such as IFN-γ. ISRE = interferon-stimulated response element, GAS = gamma interferon activation site.

Another interesting example of infection-dependent effects is IFN-α signaling. Akin to other type I IFN responses, IFN-α signaling begins with activation of a host cell pattern recognition receptor (PRR) by a pathogen. This activation leads to IRF7 phosphorylation, resulting in the upregulation of IFN-α. Once released, IFN-α binds to the interferon-α/β receptor (IFNAR), which allows IFN-α to act in an autocrine and paracrine fashion. IFNAR activation leads to the phosphorylation of STAT1 and STAT2, mediating the transcriptional upregulation of a specific set of downstream IFN-α response genes (42) (Fig. 5). As expected, the virus-infected PNCs showed upregulation in genes throughout this pathway, but the two *T. gondii* data sets were less consistent. Both *T. gondii* data sets showed an upregulation in IRF-7 but no upregulation of type I IFNs (α or β) (Fig. S4C). These findings are consistent with prior work in human fibroblasts that suggest that *T. gondii*-infected cells block type I IFN responses (33) upstream of the *T. gondii* effector TgIST, which prevents IFN signaling by binding to pSTAT1/2 heterodimers and pSTAT1 homodimers (43, 44) (Fig. 5). That the *in vivo T. gondii* data set shows the upregulation of some of the downstream IFN response genes suggests that autocrine and paracrine signaling from neuronal and non-neuronal cells may overcome this inhibition *in vivo,* or these downstream genes are upregulated by other pathways. Collectively, these findings are consistent with a model in which neuronal responses to infection depend on the context, with conserved responses arising from pathogen sensors that converge on the same downstream pathways. Such sensors could detect microbes directly or through neuronal stress that, in turn, triggers cellular responses associated with pathogen recognition (45).

## MATERIALS AND METHODS

### Parasite maintenance

As previously described, type II *T. gondii* (Pruginaud) used in this study was maintained through serial passage in human foreskin fibroblasts (gift of John Boothroyd, Stanford University, Stanford, CA) using Dulbecco’s modified Eagle medium (DMEM), supplemented with 10% fetal bovine serum (FBS), 2 mM glutagro, and 100 IU/mL penicillin and 100 µg/mL streptomycin (27).

### Mice

All mice were bred and housed in specific-pathogen-free University of Arizona Animal Care facilities. Cre reporter mice (ZsG mice) (mouse stock no. #007906) were originally purchased from Jackson Laboratories.

### Primary murine neuron culturing

Mouse primary cortical neurons were harvested from E174 mouse embryos obtained from pregnant ZsG mice. Dissections of E174 cortical neurons were performed, and primary neuronal cell cultures were generated by methods described previously with minor modifications (46). The culturing plates were prepared by coating overnight with 0.001% poly-L-lysine solution (Millipore Sigma, P4707, diluted in water 1:10) for plastic surfaces and 100 µg/mL poly-L-lysine hydrobromide (Sigma, P6282, dissolved in borate buffer, pH 8.4) for glass surfaces. They were washed three times for 10 minutes each with water and transferred to plating media (modified Eagle medium [MEM], 0.6% D-glucose, 10% FBS). Neurons were seeded at 500,000 in 6-well plates for RNA-seq. Four hours after plating, a full media exchange to neurobasal media (Neurobasal base media, 2% B27 supplement, 1% L-glutamine, and 1% penicillin-streptomycin) was performed. On day *in vitro* (DIV) 4, neurons received a half volume media change of neurobasal media with 5 µM cytosine arabinoside (AraC; Millipore Sigma, C6645) to stop glial proliferation. One-third media exchange with neurobasal media occurred every 3–4 days thereafter. All the experiments were performed on 10 DIV neurons.

### RNA isolation, preparation of cDNA libraries, and sequencing

Primary neuronal cell cultures were infected (MOI 5) for 24 hours prior to RNA isolation. Total RNA was extracted using the Direct-zol RNA Miniprep Kit and protocol (Zymo Research, R2051). Samples were sent to Novogene for quality control, library preparation, and sequencing. RNA quality was measured on an Agilent 2100. Only samples with an RNA integrity score of >7 were used. After the QC procedures, mRNA from eukaryotic organisms is enriched using oligo(dT) beads. For prokaryotic or eukaryotic organisms’ long non-coding libraries, rRNA is removed using the Ribo-Zero Kit. First, the mRNA is fragmented randomly by adding fragmentation buffer; then, the cDNA is synthesized by using mRNA template and random hexamers primer, after which a custom second-strand synthesis buffer (Illumina), dNTPs, RNase H, and DNA polymerase I are added to initiate the second-strand synthesis. Second, after a series of terminal repair, a ligation, and sequencing adaptor ligation, the double-stranded cDNA library is completed through size selection and PCR enrichment. Paired-end sequencing was performed on an Illumina NovaSeq 6000 at 20 million reads per sample. Initial QC and adapter trimming were performed by Novogene.

### RNA-seq analysis and data visualization

Analyses and visualizations were conducted as previously described (47) using a combination of statistical computing environment R version 3, RStudio version 1.2.5042, Bioconductor version 3.1 (48), and Prism 9.4.1 per a previously published training tool (49). Transcript-level counts were summarized to genes using the TxImport package (50) and mouse gene annotation package from biomaRt (51). Data were filtered and normalized with the EdgeR package (52) by the trimmed mean of *M*-values method. Genes with less than one count per million in *n* of the samples (three for PNCs and five for LCM) were filtered out. The VOOM function in Limma (53) was used to variance stabilize the filtered, normalized data. Differential gene expression analysis was performed with Benjamini-Hochberg correction with Limma (53). Gene set enrichment analysis (GSEA) was done using the GSEA software (Broad Institute, version 4.0.2) (54) in R with the GSEABase package. Venn diagrams were generated with the Venn Diagram tool from VIB/UGent Bioinformatics & Evolutionary Genomics at http://bioinformatics.psb.ugent.be/webtools/Venn/. Heatmaps were generated in R with pheatmap. Microarray data from BioProject PRJNA503843 (7), GSE122121, WNV samples GSM3455732, GSM3455733, and GSM3455734, ZKV samples GSM3455735, GSM3455736, and GSM3455737, and saline samples GSM3455737, GSM3455730, and GSM3455731 were retrieved from the GEO with the GEOquery package. The rabies data set was retrieved in the same manner, GSE38975, samples GSM953148, GSM953149, GSM953150, and GSM953151. Microarray data were analyzed with code from NCBI’s GEO2R with the Limma (53) and umap (55) packages.

## Data Availability

The data discussed in this publication have been deposited in NCBI's Gene Expression Omnibus (56, 57) and are accessible through GEO Series accession number GSE293449.

## SUPPLEMENTAL MATERIAL

Supplemental Figures msphere.00216-25-s0001.pdf Figures S1 to S4.

Table S1 msphere.00216-25-s0002.xlsx DEGs in LCM data set.

Table S2 msphere.00216-25-s0003.xlsx DEGs in PNC data set.

Table S3 msphere.00216-25-s0004.xlsx Pathways shared between LCM and PNC data sets.

Table S4 msphere.00216-25-s0005.xlsx Neuron-associated DEGs in all 4 data sets.

ASM does not own the copyrights to Supplemental Material that may be linked to, or accessed through, an article. The authors have granted ASM a non-exclusive, world-wide license to publish the Supplemental Material files. Please contact the corresponding author directly for reuse.
