# Supplementary material for: Defining neuronal responses to the neurotropic parasite Toxoplasma gondii
Source: mSphere. 2025 May 30;10(6):e00216-25. doi: 10.1128/msphere.00216-25 (PMC12188731; doi:10.1128/msphere.00216-25)
Supplement: Supplemental Figures — Figures S1 to S4. [file msphere.00216-25-s0001.pdf]

Supplemental Figures

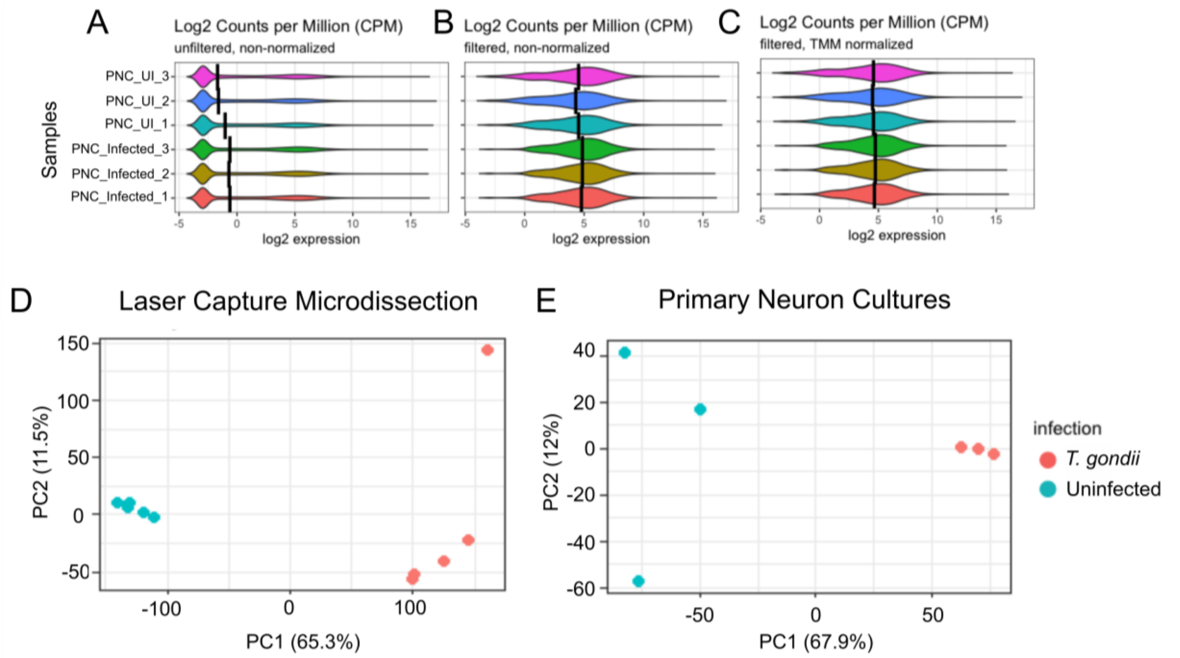

Figure S1. Normalization and data preparation in Primary Neuron Culture (PNC) analysis. (**A**) Unfiltered, non-normalized counts per million (CPM) in PNC samples. UI = uninfected. (**B**) Filtered, non-normalized CPM in PNC samples. (**C**) Filtered, TMM normalized CPM in PNC samples. (**D**) Principal component analysis of LCM. (**E**) Principal component analysis of PNC neurons.

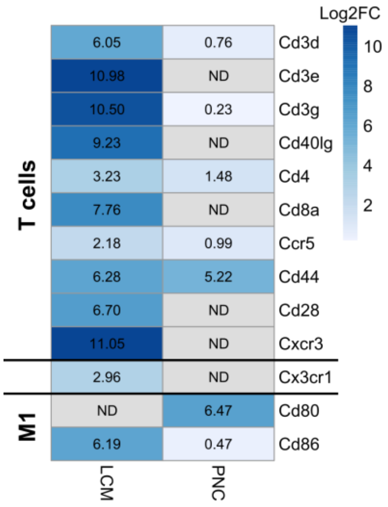

Figure S2. Heat map showing that immune cell transcripts are only observed in LCM dataset. The numbers denote the fold change between infected and uninfected samples. "ND" no differential expression between infected and uninfected datasets. *Cd44* and *Cd80* are immune cell receptors that are also expressed in neurons during development[28,29].

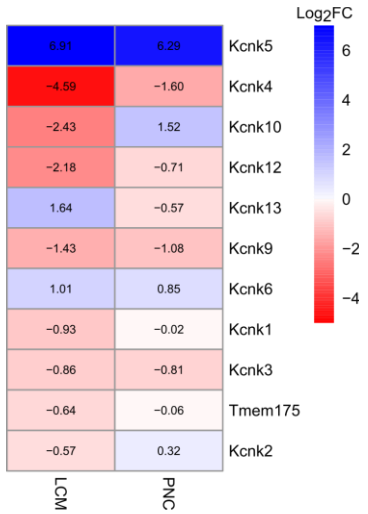

Figure S3. Potassium leak channels are downregulated in LCM and PNCs. Heatmap of genes encoding potassium leak channels shows a downregulation across *T. gondii* datasets.

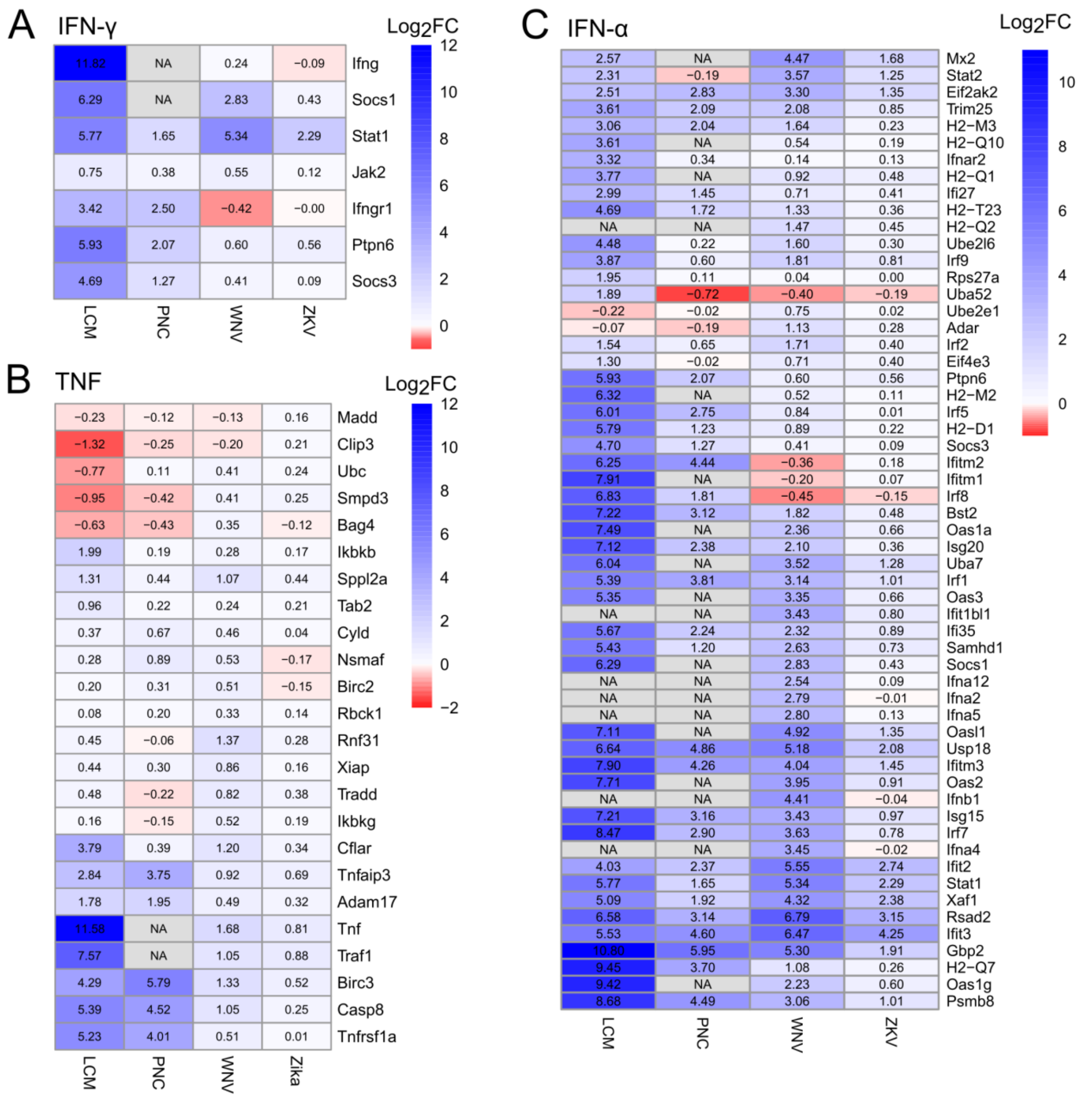

Figure S4. IFN-γ, TNF, and IFN-α pathways are differentially upregulated between datasets. (**A**) Heatmap of IFN-γ genes. (**B**) Heatmap of TNF genes. (**C**) Heatmap of IFN-α genes. Scale = Log_2FC. NA = Not Applicable/Not Detected.
